# Supplementary material for: Content-rich biological network constructed by mining PubMed abstracts
Source: BMC Bioinformatics. 2004 Oct 8;5:147. doi: 10.1186/1471-2105-5-147 (PMC528731; doi:10.1186/1471-2105-5-147)
Supplement: Additional File 5 — The original Chilibot query results of the term "long-term potentiation (LTP)" and 22 other terms, limiting the latest references analyzed to the years 1990, 1995, 2000, and 2004. [file 1471-2105-5-147-S5.bz2 › chilibotAdditionalFile5/ltp1995/html/PLC_PI-3K.html]

 


 **PLC** and **PI-3K** 
  
Found 23 abstracts in PubMed,  **23 abstracts were retrieved and analyzed**.  


---

 Search Google  |
 PDF files only 
|  EDU domain only 

---

**Interactive relationship** (e.g. stimulation, inhibition, etc)

**Parallel relationship** (e.g. studied together, co-existance, homology, etc.)

- Autophosphorylated GTEKH associated with GRB2 and SH PTP2 from endothelial lysates and not with PI3 kinase  [ **PI-3K** ]  or  **PLC**  gamma.  Ref: 7478529 Oncogene, 1995
- AG879 also inhibited NGF induced  **PLC**  gamma 1 phosphorylation, phosphatidylinositol 3 PI3 kinase  [ **PI-3K** ]  activation, the association of the tyrosine phosphorylated proteins pp100 and pp110 with the p85 subunit of PI 3 kinase  [ **PI-3K** ] , mitogen activated protein and raf 1 kinases, and c fos induction.  Ref: 7683492 Biochemistry, 1993
- There was no difference in starved or nonstarved cells regarding  **PLC**  gamma activation, increase of calcium i, and stimulation of PI 3 kinase  [ **PI-3K** ]  activity.  Ref: 7962118 J Cell Physiol, 1994
- These results indicate that tyrosine phosphorylation of  **PLC**  gamma 1, GAP, and PI 3 kinase  [ **PI-3K** ]  are specific responses for VSMC hyperplasia but not thromboxane stimulated hypertrophy.  Ref: 7810610 Am J Physiol, 1994
- Further studies show that PDGF induced tyrosine phosphorylation of phospholipase C gamma 1  **PLC**  gamma 1 and phosphatidylinositol 3 kinase PI 3 kinase  [ **PI-3K** ]  are also suppressed in dibutyryl cAMP pretreated cells.  Ref: 7790382 J Cell Physiol, 1995
- The tyrosine phosphorylation or activity of several cellular proteins, such as  **PLC**  gamma 1, PI 3 kinase  [ **PI-3K** ] , and Erk1 and the expression of the mRNA for the late response gene transin were also sustained as a consequence of gp140trk overexpression.  Ref: 1384575 Neuron, 1992
- Finally, beta PDGF receptors that have tyrosine to phenylalanine point mutations at positions 70 1 77 and 989 did not associate with either PI 3 kinase  [ **PI-3K** ]  or  **PLC**  gamma 1.  Ref: 7743180 Curr Biol, 1995
- In contrast, similar treatment with normal human astrocytes potentiates the tyrosine phosphorylation of  **PLC**  gamma 1 and PI 3 kinase  [ **PI-3K** ] .  Ref: 7790382 J Cell Physiol, 1995
- Primary targets within 10 15 minutes were identified as PI 3 kinase  [ **PI-3K** ] , GAP,  **PLC**  gamma, src, and MAP kinase, substrates which are also activated upon growth factor activation of mitogenic receptor systems.  Ref: 8380734 EXS, 1993
- However, no other downstream responses to NGF stimulation such as tyrosine phosphorylation of  **PLC**  gamma 1, PI 3 kinase  [ **PI-3K** ] , ERK1 and ERK2, induction of FOS and NGFI A mRNAs, and neurite extension were observed in the unresponsive cell lines.  Ref: 7972209 Prog Clin Biol Res, 1994
- In Western analysis of phosphotyrosine immunoprecipitates, the hyperplastic agents PDGF and PMA increased tyrosine phosphorylation of phospholipase C gamma 1  **PLC**  gamma 1, GTPase activating protein GAP, and phosphatidylinositol 3 kinase PI 3 kinase  [ **PI-3K** ] , while the hypertrophic agonist thromboxane failed to tyrosine phosphorylate either of these three substrates.  Ref: 7810610 Am J Physiol, 1994
- PC12 cells expressing the beta PDGF receptor extend neurites in response to PDGF in the absence of signalling through PI 3 kinase  [ **PI-3K** ] , RasGAP, and  **PLC**  gamma 1.  Ref: 7743180 Curr Biol, 1995
- The discovery that rasGAP, PI 3 kinase  [ **PI-3K** ]  and  **PLC**  gamma 1 are substrates for the EGF receptor tyrosine kinase has provided a beginning in understanding the biochemistry underlying growth factor receptor transduction.  Ref: 1501243 J Membr Biol, 1992
- CD40 engagement also resulted in phosphorylation of phospholipase C gamma 2 of phosphatidylinositol  **PLC**  gamma 2 and phosphatidylinositol PI 3 kinase  [ **PI-3K** ] .  Ref: 7507510 J Exp Med, 1994
- METHODS Immunodetection was used to compare the expression and tyrosine phosphorylation state of  **PLC**  gamma 1, EGFR, phosphatidylinositol 3 kinase PI 3 kinase  [ **PI-3K** ] , ras guanosine triphosphatase activating protein GAP, and src homologous collagen like protein SHC in the postnatal rat intestine.  Ref: 8020653 Gastroenterology, 1994
- To transmit NGF signals, NGF activated Trk rapidly associates with the cytoplasmic proteins, SHC, PI 3 kinase  [ **PI-3K** ] , and  **PLC**  gamma 1.  Ref: 7852994 J Neurobiol, 1994
- Tyrosine kinase inhibition with herbimycin A 5 microM prevented agonist stimulated tyrosine phosphorylation of  **PLC**  gamma 1, GAP, and PI 3 kinase  [ **PI-3K** ] .  Ref: 7810610 Am J Physiol, 1994
- Three signaling pathways were studied in detail 1 Phospholipase C gamma  **PLC**  gamma and calcium i increase, 2 activation of the phosphatidylinositol 3 kinase PI 3 kinase  [ **PI-3K** ] , and 3 activation of mitogen activated kinases I and II MAP kinases I and II.  Ref: 7962118 J Cell Physiol, 1994
- The resulting constrained pTyr mimetics were examined for inhibitory potency in six SH2 domain constructs Lck, Src, Grb2, and the C terminal SH2 domains of  **PLC**  gamma  **PLC**  gamma C and the p85 subunit of PI 3 kinase  [ **PI-3K** ]  p85 C, as well as the N terminal SH2 domain of SH PTP2.  Ref: 7537333 J Med Chem, 1995
- These results suggest that lyn plays an important role in CD40 mediated PTK activation and identify  **PLC**  gamma 2 and PI 3 kinase  [ **PI-3K** ]  targets for CD40 mediated phosphorylation, suggesting a role for these two enzymes in CD40 signal transduction.  Ref: 7507510 J Exp Med, 1994
